# Enriched marine oil supplements in pregnancy for the modulation of maternal inflammatory- associated causes of preterm delivery

**DOI:** 10.12688/f1000research.153569.2

**Published:** 2024-10-31

**Authors:** Pedro Antonio Regidor, Johanna Eiblwieser, Theresa Steeb, Jose Miguel Rizo

**Affiliations:** 1 Medical Department, Exeltis Healthcare, Ismaning, 85737, Germany; 2 Medical Department, Exeltis Germany, Ismaning, Adalperostr. 84, 85737, Germany; 3 Chemo OTC, Madrid, Madrid, 28050, Spain

**Keywords:** pregnancy; specialized pro-resolving mediators; polyunsaturated fatty acids; chronic inflammation; preterm birth; preeclampsia; amniotic inflammation; chorioamnionitis

## Abstract

Preterm birth is a major cause of perinatal complications and neonatal deaths. Furthermore, in the field of obstetrics many clinical entities like uterine contractions or the occurrence of pre- eclampsia remain to be serious complications during pregnancy and represent a major psychological, financial, and economic burden for society. Several published guidelines, studies and recommendations have highlighted the importance of supplementation of omega-3 long chain polyunsaturated fatty acids (PUFAs) during pregnancy. This narrative review aims at giving an overview on the modern perception of inflammatory processes and the role of specialized pro-resolving mediators (SPMs) in their resolution, especially in obstetrics. Additionally, we highlight the possible role of SPMs in the prevention of obstetric complications through oral supplementation using enriched marine oil nutritional’s. The intake of PUFAs may result in an overall improvement of pregnancy outcomes by contributing to fetal brain growth and neurological development but more importantly though modulation of inflammation-associated pathologies. Especially the use of SPMs represents a promising approach for the management of obstetric and perinatal complications. SPMs are monohydroxylates derived from enriched marine oil nutritional’s that involve certain pro-resolutive metabolites of omega-3 long chains PUFAs and may contribute to an attenuation of inflammatory diseases. This may be obtained through various mechanisms necessary for a proper resolution of inflammation such as the termination of neutrophil tissue infiltration, initiation of phagocytosis, downregulation of pro-inflammatory cytokines or tissue regeneration. In this way, acute and chronic inflammatory diseases associated with serious obstetrical complications can be modulated, which might contribute to an improved pregnancy outcome.

## Plain language summary

Obstetric and perinatal complications such as preterm birth or pre-eclampsia represent a serious clinical issue. The supplementation of omega-3 long chain polyunsaturated fatty acids (PUFAs) during pregnancy might have the potential to improve pregnancy outcomes by modulation of inflammatory processes. Specifically, the use of selective pro-resolving mediators (SPMs), including monohydroxylates derived from omega-3 long chain PUFAs, can potentially modulate acute and chronic diseases, particularly in obstetrics. Enriched marine oil nutritional’s containing SPMs might therefore aid in the resolution of inflammation and potentially improve pregnancy outcomes. This narrative review provides a summary of the current understanding of inflammatory processes and the importance of SPMs in resolution of inflammation, with a focus on obstetrics.

## 1. Introduction: The burden of preterm birth

A major cause of more than 85% of all perinatal complications and neonatal deaths is preterm birth, mainly early preterm birth ( Ward and Beachy 2003, D’Apremont *et al.* 2020). Children born prematurely often suffer from a variety of physical and neurodevelopmental disorders, which may cause substantial consequences ( Ward and Beachy 2003). Among these are for example neurocognitive complications, which can occur early in childhood and manifest as developmental delay, cerebral palsy, hearing, and visual impairments, learning difficulties and psychiatric disorders ( Saigal and Doyle 2008). Neurodevelopmental disabilities and recurring health issues have a significant impact during early childhood, resulting in the appearance of less conspicuous disabilities such as learning difficulties or behavioral issues which continue until adolescence ( Saigal and Doyle 2008). Furthermore, research consistently demonstrates that adults who were born preterm face elevated risks of chronic conditions affecting multiple organ systems, including cardiovascular, endocrine/metabolic, respiratory, renal, neurodevelopmental, and psychiatric issues. These conditions may persist from childhood or only become apparent in adulthood. Additionally, individuals born preterm experience moderately higher mortality risks (30% to 50%) in early to mid-adulthood compared to those born full-term, with even greater risks observed in those born at the earliest gestational ages ( Crump 2020). Moreover, preterm birth imposes significant psychological and financial burdens on parents. With estimated costs of $1.6 billion to $26 billion annually in the United States, preterm birth represents a considerable economic burden and impact on society ( Institute of Medicine (US) Committee on Understanding Premature Birth and Assuring Healthy Outcomes 2007, Waitzman *et al.* 2021, Newnham *et al.* 2022). Nevertheless, factors like the country, population size, and preterm birth rate have a strong influence on the range of costs ( Institute of Medicine (US) Committee on Understanding Premature Birth and Assuring Healthy Outcomes 2007, Waitzman *et al.* 2021, Newnham *et al.* 2022). Although a large portion of these costs are attributable to neonatal intensive care for infants born very prematurely, further additional costs have been shown to be associated with prematurity. These costs extend beyond the initial hospitalization, even for children born only a few weeks prematurely. However, two thirds of preterm births occur without known biological causes. Hence, strategies for prevention are urgently warranted and still a subject of intense debate ( Ferrero *et al.* 2016, Goodfellow *et al.* 2021).

## 2. Supplementation with polyunsaturated fatty acids (PUFAs) to prevent preterm birth

Notably, maternal supplementation with omega-3 long chain polyunsaturated fatty acids (long chain PUFAs) might be one of the most promising interventions to prevent preterm (<37 weeks gestation) and early preterm (<34 weeks gestation) birth ( Middleton *et al.* 2018, Makrides et al. 2019, Carlson *et al.* 2021). Therefore, an assessment of the need for omega-3 long chain PUFAs during pregnancy and their specific role in reducing the risk of preterm birth is needed. Most guidelines and dietary advice suggest screening and adjust dietary habits to achieve an adequate supply of omega-3 long chain PUFAS, particularly docosahexaenoic acid (DHA) and eicosapentaenoic acid (EPA) ( Koletzko *et al.* 2007, 2008). Recommendations include that pregnant and lactating women should aim to achieve a dietary intake of omega 3 PUFA that supplies a DHA intake of at least 200 mg/day and that women of childbearing age can meet the recommended intake of DHA by consuming one to two portions of sea fish per week, including fatty fish, which is a good source of omega-3 long chain PUFA ( Koletzko *et al.* 2007, 2018, Coletta *et al.* 2010). Notably, DHA and EPA have been shown to contribute to myometrial relaxation and thus might prevent early onset of labor. Additionally, they may also inhibit the activation of trophoblastic inflammatory pathways. This could lead to a decrease in inflammation-associated preterm birth ( Frew *et al.* 2013, Jones *et al.* 2014). Furthermore, they have been associated with maintaining the fetal supply of omega-3 long chain PUFAs and thus, support brain growth and subsequent neurological development in infants and children ( Koletzko *et al.* 2007, 2008).

However, many of the recommendations and guidelines, for example the consensus statement of the Perinatal Lipid Working Group supported by the International Society for the Study of Fatty Acids and Lipids (ISSFAL) which has been published in 2007, have not been updated for years. Furthermore, none of them specifically addresses the impact of omega-3 long chain PUFAs on prematurity ( Koletzko *et al.* 2007).

The interest in maternal dietary intake of omega-3 long chain PUFAs and perinatal outcomes has increased over the previous years. Until now, several reviews from the Cochrane Collaboration and an abundance of randomised controlled trials have been published on omega-3 long chain PUFAs and preterm birth, highlighting the importance of this subject. The most recent Cochrane review from 2018 has synthesised evidence and included 70 randomised controlled trials (involving 19,927 women at low, mixed or high risk of poor pregnancy outcomes) ( Middleton *et al.* 2018). In 2021, this Cochrane analysis has been updated by Best et al., who further involved crucial trials which have been conducted since. Consequently, a total of 80 clinical trials could be included and omega-3 long chain PUFA supplementation and risk of preterm birth was evaluated more thoroughly in the “2021 Cochrane update” ( Best *et al.* 2022). The analysis revealed improvements in several outcomes such as a longer duration of gestation (1-2 days), higher birth weight and reduced risks of preterm birth and early preterm birth associated with omega-3 long chain PUFAs ( Middleton *et al.* 2018). In summary, the results suggest that there is high-quality evidence that supplementation with omega-3 long chain PUFAs during pregnancy, particularly with DHA, reduced the risk of having a premature baby born before 37 weeks’ gestation, by 12% (RR 0.88; 95% confidence interval [CI] 0.81 – 0.95) and it also reduced the risk of having a very premature baby, born before 34 weeks, by 35%, (RR 0.65; 95% CI 0.46 – 0.92) in comparison with no or only minimal omega-3 fatty acid intake. Furthermore, also other parameters associated with prematurity were evaluated: It could be demonstrated that birthweight will also be affected by omega-3 long chain PUFAs. Analysis of the data in the 2021 Cochrane update showed a mean increase of 71 g (95% CI 39 –102 g). No association with small for gestational age (RR 1.02; 95% CI 0.93 – 1.13) was found and a potential increase in large for gestational age (RR 1.13; 95% CI 1.01 – 1.28) ( Best *et al.* 2022, Middleton *et al.* 2018).

The recommended doses for omega-3 long chain PUFA might vary regarding the time of pregancy: Larger reductions in preterm births before 37 weeks of gestation were observed with the 500–1000 mg dose, while doses above 1000 mg showed the greatest effect in reducing early preterm births before 34 weeks of gestation ( Best *et al.* 2022).

These assumptions are supported by various studies. In a case-control study, Olsen et al. observed an elevated risk of early preterm birth in women with low plasma levels of omega-3 PUFA ( Olsen *et al.* 2018, 2020). Carlson *et al.* conducted a randomized, double-blind superiority trial, in which the group hypthesized a dose of 1000 mg DHA to be superior to 200 mg. They recognized fewer serious adverse events sich as chorionamnionitis, premature rupture of membranes and early preterm brith in women taking the higher dose. The authors assume an antiinflammatory effect which might prevent early labor ( Carlson *et al.*, 2021). In another trial by Simmonds et al., the study group received ~ 900 mg omega-3 PUFA per day, while the control group supplemented only vegetable oil with traces of fish oil (~ 20 mg/day). Women with a high omega-3 PUFA intake were found to have a substantially decreased risk of early preterm birth compared to controls with low omega-3 PUFA. Thus, women who have low levels of total omega-3 PUFAs in early pregnancy face a higher risk of early preterm birth and are the most likely to benefit from omega-3 supplementation to lower this risk. On the other hand, women with higher omega-3 levels are at a reduced risk, and further omega-3 supplementation may actually increase their risk ( Simmonds *et al.* 2020).

Therefore, the authors concluded that pregnant women with one baby should be advised to take between 500 and 1000 mg of long- chain omega-3 PUFAs every day from the 12th week of pregnancy, to increase their chances of having a healthy full-length pregnancy ( Middleton *et al.* 2018).

## 3. The role of lipid derived mediators in initiation and resolution of inflammation

**Figure 1.**
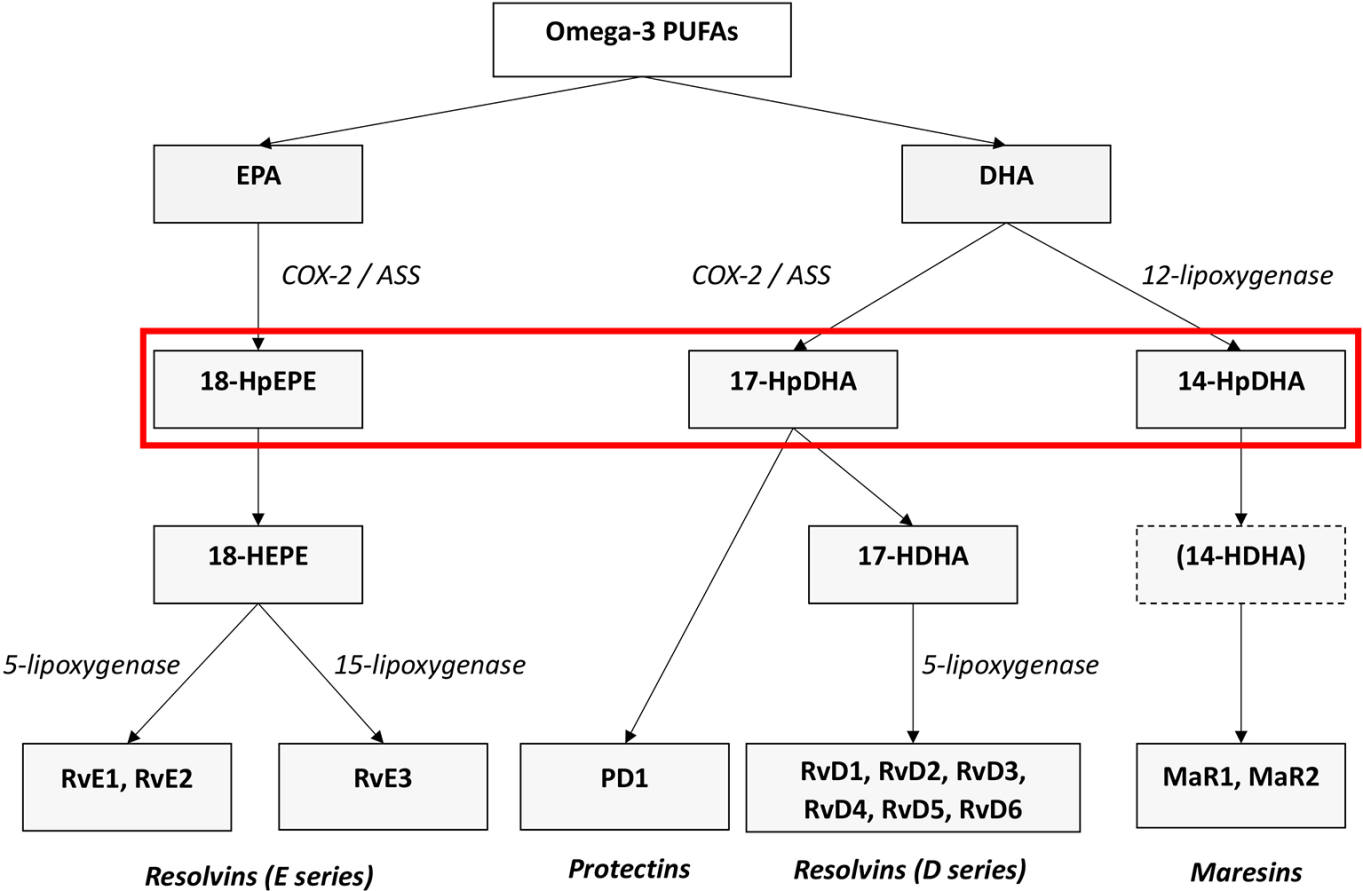
Biosynthesis of the SPMs Resolvins, Protectins and Maresins. EPA; Eicosapentaenoic Acid; 18-HpEPE, 17-HpDHA, 14-HpDHA (highlighted by the red box): precursors of the SPMs during biosynthesis; Cox-1/2: Cyclooxygenases. LOX: lipoxygenase. ASS: aspirin. Aspirin triggers biosynthesis of 18-HpEDE, 17-HpDHA intermediates via modification of COX-enzymes. Maresins are produced by macrophages via a preliminary lipoxygenation step. Further lipoxygenases are required for SPM biosynthesis as depicted (modified from Serhan 2017).

A microbial infection or injury usually leads to acute inflammation with the aim of eliminating pathogens, removing cellular debris and finally restoring affected tissue. Important pro-inflammatory signalling molecules include eicosanoid lipid mediator molecules which are synthesized from the omega-6 PUFAs arachidonic acid (AA). These prostanoids comprise the prostaglandins (PG), leukotrienes (LT) and thromboxanes (TX). They are synthesized via the enzymes cyclooxygenases 1 and 2 (COX-1 and -2) by cells of the innate immune system, i.e. granulocytes or macrophages which are immediately attracted to the localisation of the respective injury or infection ( Chiurchiu *et al.* 2018). Mast cells secret further pro-inflammatory cytokines such as tumor necrosis factor-alpha (TNFα) and interleukins 1 and 6 (IL-1 and IL-6). Besides, other pro-inflammatory cytokines such as IL-1β, IL-12 and IL-18 are produced by M1 macrophages ( Chavez-Galan *et al.* 2015). Thus, an inflammatory response is evoked which is characterized by the cardinal symptoms of inflammation such as heat (calor), swelling (tumor), redness (rubor), pain (dolor), and potentially a loss of function (functio laesa). Neutrophils and monocytes are also attracted to the site of infection of injury by leukotriene B4 (LTB4) and cytokines. Following this, they infiltrate the affected tissue, and thus, advance the organisms’ inflammatory response ( Flower 2006, Samuelsson 2012, Chiurchiu *et al.* 2018). As the initiation of inflammation is essential to provide immediate response and limitation to entry of pathogens, remove damaged cells and enable quick tissue regeneration, it should be triggered rapidly and efficiently. However, on the same time, the cessation of inflammation is equally important and should be quickly initiated as well to prevent further impairment of the organism. Importantly, life-threatening events like cytokine storm or sepsis may occur following excessive, unlimited inflammation ( Hotchkiss *et al.* 2016). Mediation and resolution of inflammation is an active process triggered by specialized pro- resolving lipid mediator molecules (SPMs), which has been shown in animal models and in different human cells ( Serhan *et al.* 2000, Serhan 2014). They are categorized into four families according to their chemical structure and biosynthetic pathways: D- and E-resolvins (RvD and RvEs, protectins (PD), lipoxins (LX) and maresins (MaR) ( Serhan 2014, Serhan *et al.* 2000, 2015). RvD, PD, and MaR derive from the omega-3 PUFA DHA, RvE originate from EPA (see Figure 1), and LX stem from the omega-6 AA. Lipoxygenases as well as COX- enzymes are part of the biosynthesis of the SPMs. SPMs are synthesized via the hydroxylated precursors 18-HpETE, 17-HpDHA, and 14-HpDHA ( Serhan *et al.* 2015, Serhan 2017). Aspirin irreversibly binds COX enzymes and thus blocks the synthesis of prostanoids by altering the catalytic domains of COX. However, their capacity to catalyze the synthesis of the SPM precursors 18-HpETE, 17-HpDHA and 14-HpDHA is not abolished. The newly formed SPMs have a different stereochemical structure and are known as aspirin-triggered SPMs (AT-SPMs) ( Serhan *et al.* 2000, 2002, 2011, 2015) (see Figure 1).

**Figure 2.**
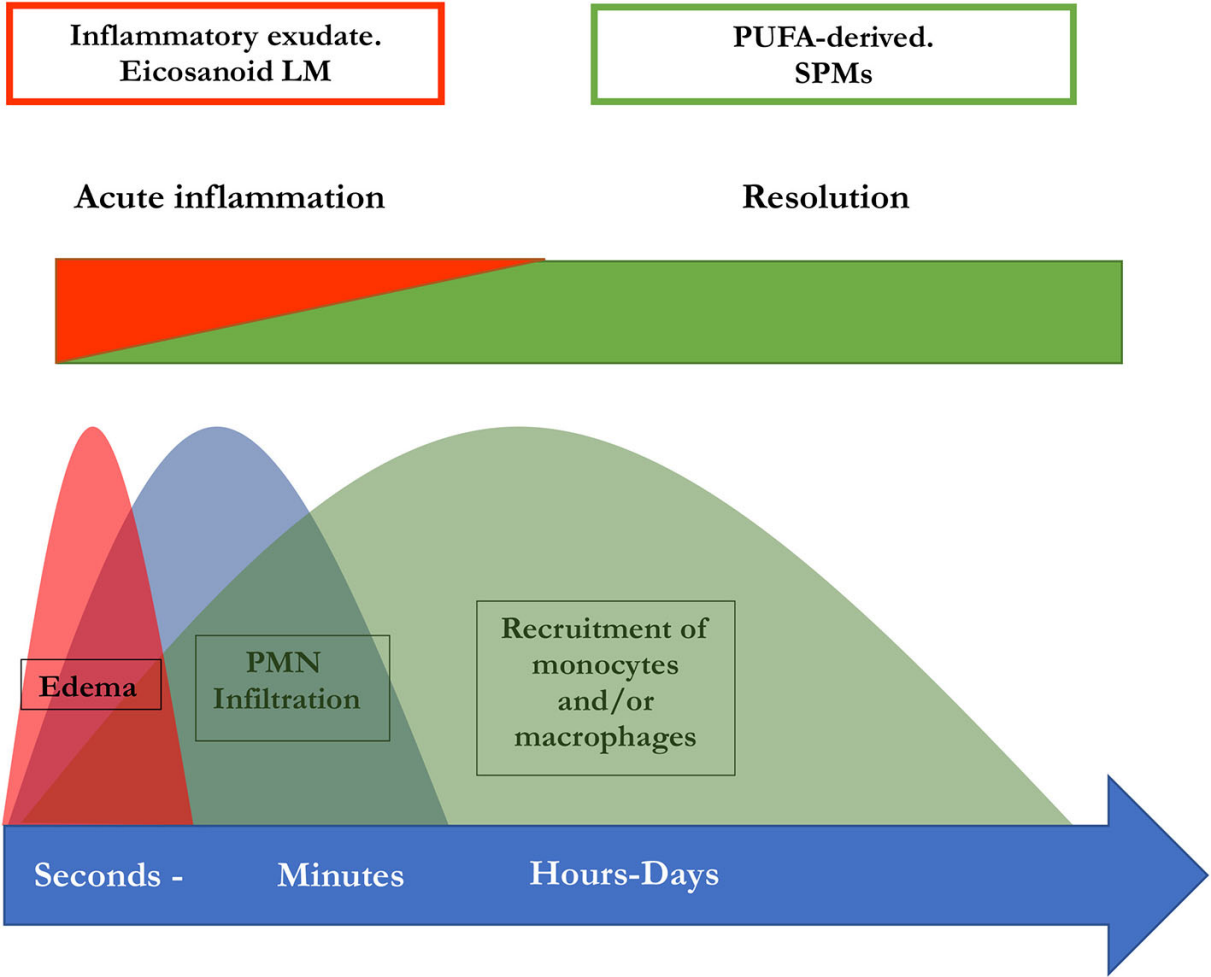
Concomitant initiation and resolution of inflammation. Synthesis of pro-resolving lipid mediator molecules is initiated in the beginning of inflammatory processes. LM: Lipid mediator; PUFA: poly-unsaturated fatty acid, PMN: polymorphonuclear neutrophils; SPM: specialized pro-resolving mediators (maresins, resolvins, protectins, and lipoxins) (modified from Serhan and Levy 2018).

Several key steps are responsible for the termination of inflammatory processes: dead cells have to be removed, a conversion of macrophages to resolving M2 macrophages must be initiated, and the recruitment of neutrophils must be stopped ( Serhan *et al.* 2002, Serhan and Chiang 2013, Serhan 2017, Schett and Neurath 2018, Serhan and Levy 2018). SPMs have a vital role in the termination of neutrophil infiltration and in the initiation of phagocytosis of apoptotic cells. Besides, they are involved in the downregulation of pro-inflammatory cytokines, such as TNF-α, IL-6, IL-8 and IL-12, the reduction of platelet-activating factor and prostaglandin production. In addition to that, SPMs are also involved in the clearance of the infection site and tissue regeneration, by stimulating efferocytosis and phagocytosis, promoting wound healing. These resolutive processes are triggered simultaneously to inflammation which is evident from the interlinkage of prostaglandin synthesis with the SPM biosynthetic pathways. Both the generation of pro-inflammatory lipid mediators and the subsequent synthesis of inflammation- mediating SPMs are promoted by polymorphonuclear leukocytes (PML) ( Levy *et al.* 2001). This implies a lipid mediator class switch in these cells which is essential for a regulated resolution of inflammation and thus for the prevention of chronification ( Levy *et al.* 2001). PGE2 as well as PGD2 are necessary to induce lipoxygenases which are in turn necessary for the generation of LXs, Rvs, and Protectin D1 ( Serhan and Savill 2005). Therefore, the switch of the lipid mediator class from a proinflammatory to a pro-resolutive function is disrupted when PG synthesis is inhibited. Hence, this may result in impaired resolution ( Bandeira-Melo *et al.* 2000, Levy *et al.* 2001). In summary, the initiation of inflammation is inseparably linked to its active resolution, thus the beginning of the signaling cascade programs the end ( Serhan and Savill 2005). An illustration of this perception of inflammation is available in Figure 2.

Although the ability of pro-resolution remains to be the most important activity, SPMs also have other effects related to adaptive immune response. LX also triggers the activation of natural killer cells ( Barnig *et al.* 2013). CD4+ T cell differentiation has also been shown to be modulated by the resolvins RvD1, RvD2 and maresin MaR1 ( Chiurchiu *et al.* 2016). Notably, 17-HDHA and RvD1 increased IgM and IgG production in human B cells, suggesting SPM activity in humoral response and opening new functions as endogenous non-toxic adjuvants ( Ramon *et al.* 2012).

## 4. The significance of SPMs in chronic inflammatory diseases

**Figure 3.**
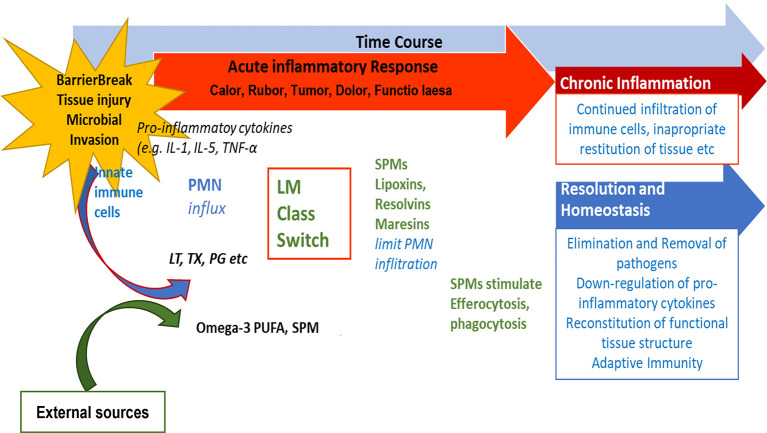
Role of SPMs during resolution of inflammation and potential outcome of inflammatory processes. After an inflammation is triggered, cells of the innate immune system synthesize eicosanoid lipid mediator (LM) molecules (LT, TX, PG etc) from AA, and pro-inflammatory cytokines that stimulate the influx of PMN and monocytes and result in the classical signs of acute inflammation ( *calor*, *rubor*, *tumor*, *dolor*, *functio laesa*). Already in the beginning, eicosanoids trigger the LM class switch resulting in the synthesis of SPMs from omega-3-PUFAs. SPMs are crucial for resolution of inflammation. LM: Lipid mediator; LT: Leukotriene, TX: Thromboxane, PG: Prostaglandins; PUFA: poly-unsaturated fatty acid, SPM Specialized pro-resolving mediators (modified from Serhan 2017).

Previous and ongoing research on SPMs has increased the knowledge on their structure and their biosynthetic pathways, receptors and function. Serhan *et al.* ( Serhan 2014, 2017, Serhan *et al.* 2015) and Chiang *et al.* have suggested different options for the outcome of inflammatory responses ( Chiang *et al.* 2019). If inflammation does not resolve sufficiently and remains active, i.e., pro-inflammatory signaling molecules are constantly produced, a state of chronic inflammation may develop and the activation of COX-2 enzymes is induced ( Serhan *et al.* 2003). This is depicted in Figure 3.

In air pouch mouse models, researchers mimicked this situation successfully. Another animal model revealed the role of the SPMs LXA4, RvD1 and RvD2 in the pathogenesis of atherosclerosis, a chronic inflammatory disease ( Merched *et al.* 2008). In mice transgenic for 12-/15-lipoxygenase, increased expression of RvD1, PD1, and 17-HpDHA proved to be protective by reducing the development of atherosclerosis compared to wild-type mice. The anti-atherogenic effects of LXA4, PD1, and RvD1 rely on several processes, including decreased expression of endothelial adhesion molecules and decreased secretion of cytokines. Importantly, the influence of nutrition on the pathogenesis of atherosclerosis has been shown in this mouse model as the transgenic mice were as susceptible to atherosclerosis as the wild type animals following a standard high- fat western diet ( Merched *et al.* 2008, Merched *et al.* 2011). Furthermore. experiments in a rat model for arthritis have been conducted which demonstrated that RvD1 and the precursor metabolite 17-HDHA reduced pain and tissue damage more effectively in comparison to steroids ( Lima-Garcia *et al.* 2011).

Fibrosis represents one of the characteristic features of uterine leiomyoma ( Food & Agriculture Organization of the United Nations (FAO), 2010). Thus, inadequate resolution of inflammation might be considered as a cause for uterine fibroids (UF) or uterine leiomyoma. Recent animal models revealed various insights into the role of resolution of inflammation in fibrosis: For example, an animal model in pulmonary fibrosis demonstrated that exogenously administered LX4-epimer (AT-LX4) reduced fibrosis ( Martins *et al.* 2009). Additionally, LXA4 and its analogue benzo-LXA4 reduced the extent of fibrotic changes in kidney in a rat model of early renal fibrosis, as well ( Borgeson *et al.* 2011). A mouse model of obstructed kidney showed anti- fibrotic effects for RvE1 ( Qu *et al.* 2012).

To date, the role of SPMs in the development of UF has not yet been investigated. However, there are some features that UF has in common with other chronic inflammatory diseases where consensus has been reached on the importance of inadequate resolution, including for example the role of SPMs in tumours ( Fishbein *et al.* 2021). Still, further research in this context is urgently warranted and might lead to novel therapeutic options and insights.

## 5. SPMs in intraamniotic inflammation and clinical chorioamnionitis

In patients in preterm delivery with intact chorioamniotic membranes as well as in patients experiencing prelabor rupture of membranes, intraamniotic inflammation can lead to an intense systemic maternal inflammatory response which is described as clinical chorioamnionitis ( Gravett *et al.* 1986, Gibbs *et al.* 1992, Romero *et al.* 2014, 2016a). This condition ranks among the most prevalent infection-associated diseases globally, primarily affecting young primiparous women ( Malloy 2014). The prevalence of chorioamnionitis in the United States was 9.7 per 1000 live births in 2008 ( Romero *et al.* 2015). Apart from systemic inflammatory symptoms in the mother, also acute symptoms of histologic chorioamnionitis have been reported ( Kim *et al.* 2015) as well as inflammatory responses affecting the fetus which are associated with funisitis or chorionic vasculitis ( Romero *et al.* 2007a, 2007b, Christiaens *et al.* 2008). Besides being linked to maternal morbidity, neonates which are delivered by mothers suffering from clinical chorioamnionitis at term (TCC) possess an enhanced risk for long-term consequences such as cerebral palsy ( Becroft *et al.* 2010, Elovitz *et al.* 2011, Thomas and Speer 2011). TCC is currently described as a heterogenous disease pattern which is accompanied by symptoms like fever, leukocytosis, foul-smelling amniotic fluid, maternal or fetal tachycardia or uterine tenderness ( Newton 1993, Tita and Andrews 2010, Romero *et al.* 2016b). A study evaluating patients suffering from clinical chorioamnionitis reported 24% with sterile intra-amniotic inflammation and 54% with microbial-associated intra-amniotic inflammation ( Romero *et al.* 2015). To unravel the causal mechanisms of a sterile inflammatory response, studies could demonstrate the influence of damage-associated molecular patterns (DAMPs) on sterile inflammation, for example the high mobility gene box-1 ( Chen and Nunez 2010). Furthermore, it could be shown that the amniotic fluid in TCC-patients has high DAMP-levels ( Romero *et al.* 2012) which are also associated with induction of labor ( Gomez-Lopez *et al.* 2016). The introduction of clinical tests which can assess the differential diagnosis of the three distinct subgroups of chorioamnionitis-patients, i.e. acute, chronic or subclinical chorioamnionitis, would be a helpful tool since these patient cohorts need different therapy approaches. Patients diagnosed with microbial-associated intra-amniotic inflammation require antibiotics while patients without any intra-amniotic inflammation do not. The establishment of a clinical biomarker for the detection and identification of chorioamnionitis regardless of the presence of an intra-amniotic infection would contribute to an improved characterization and diagnosis of this disease.

SPMs play a crucial role in the mediation and resolution of microorganism-derived and sterile inflammation ( Harris *et al.* 2002, Serhan and Savill 2005, Serhan *et al.* 2008a, Ricciotti and FitzGerald 2011). Since it has been recognized that PGs such as PGE2 and LTs such as LTB4 are elevated in the amniotic fluid in clinical chorioamnionitis, an important contribution of these bioactive lipids in delivery at term can be assumed. Additionally, patients with microbial- associated intra-amniotic inflammation together with clinical chorioamnionitis show significantly high concentrations of AA-derived SPMs compared to those with sterile intra- amniotic inflammation ( Maddipati *et al.* 2016a). Moreover, since TCC is characterized as an acute inflammatory condition, it is assumed that its lipid profile in the amniotic fluid differs from spontaneous labor at term (TLB). In this context, it could be demonstrated that there is no difference between concentrations of proinflammatory lipids in amniotic fluid in TLB and TCC patients. However, in all patients with TCC the presence of SPMs was significantly reduced compared to TLB patients, suggesting a decreased synthesis of SPMs as a characteristic property of TCC as opposed to infection-driven intra-amniotic inflammation where lipid mediators play an essential role ( Maddipati *et al.* 2016b).

## 6. Pre-eclampsia

Hypertension, in particular pre-eclampsia (PE), which accounts for most fetal, neonatal and maternal deaths, is one of the most common complications worldwide during pregnancy ( Redman and Sargent 2005). Women who survive pre-eclampsia tend to have shorter life expectancies, facing higher risks of stroke, heart disease, and diabetes. Resolution of PE can be achieved through mostly pre-term delivery of the baby and the placenta. Babies born from pre-eclamptic pregnancies are more likely to experience premature birth (often medically indicated), as well as death around the time of birth, developmental disorders, as well as long-term cardiovascular and metabolic health issues ( Dimitriadis *et al.* 2023). Possible symptoms manifest after the 20th week of pregnancy and include de novo hypertension, edema, and proteinuria ( Saftlas *et al.* 1990, Redman and Sargent 2005, Young *et al.* 2010). The exact mechanisms underlying the cascade may prompt an endothelial response within the vasculature ( Redman and Sargent 2005) wall as the role of pro-inflammatory pattern-recognition receptors ( Sado *et al.* 2011). Despite these research efforts, the precise mechanisms by which inflammation influences the pathogenesis of PE remains incompletely understood.

All pregnant women show a systemic inflammation evoked though clearance of placental debris which is released into the maternal blood circulation. PE occurs simultaneously when a failure of the systemic inflammatory response decompensates the immunogenic burden ( Redman and Sargent 2001). Due to this correlation, it can be assumed that an impairment of anti- inflammatory processes is crucial for the development of a PE in pregnant women. In this context, SPMs act as potentially important “braking signals” of inflammation ( Schwab and Serhan 2006, Serhan *et al.* 2008b, Maderna and Godson 2009). Even if findings from clinical studies investigating the benefit of omega-3 supplementation during pregnancy for the prevention of PE are limited, it could be shown that their status seems to be compromised during this disease ( Burchakov *et al.* 2017). Moreover, the supplementation of EPA, DHA and/or alpha-linoleic acid (ALA) during PE or pregnancy-induced hypertension has been demonstrated to yield favorable effects. A meta-analysis of 14 clinical trials, either placebo-controlled or compared with another supplementation, revealed that supplementation of n-3 fatty acids or their metabolites plays a protective role to prevent the risk of PE in women with low-risk pregnancies ( Bakouei *et al.* 2020).

The SPM LXA4 for example is able to bind to the G-protein-coupled receptor N-formyl peptide receptor 2 (FPR2/ALX) (11) whose downregulatory signalling contributes fundamentally to LXA4-mediated anti-inflammatory response in vivo. Thereby, LXA4 acts as an inhibitor of chemotaxis of eosinophilic and neutrophilic granulocytes ( Takano *et al.* 1997, Serhan 2007), antagonizes peptido-LTs ( Badr *et al.* 1989), enhances macrophage-mediated phagocytosis of apoptotic cells ( Maderna and Godson 2009), and reduces neutrophile infiltration in vivo ( Takano *et al.* 1997). Since LXA4 is a crucial regulator of inflammatory response, a contribution to PE can be assumed. This was investigated by Dong et al., who quantified the expression of LXA4, TNF-α and IL-1β in the peripheral blood of pregnant women suffering from different degrees of PE. It was found that throughout progression of PE, LXA4 increases but less than either TNF- α or IL-1β. However, no effect on the fetus could be observed. The ratio of LXA4 to TNF-α and IL-1β showed a significant reduction, resulting in a relative decrease in LXA4 levels. Interestingly, no LXA4 was detected in the umbilical cord blood of either healthy individuals or those with pre-eclampsia. Additionally, the study observed an increase in LXA4 receptor FPR2/ALX mRNA in placentas affected by pre-eclampsia ( Dong and Yin 2014). On the other hand, a study conducted by Xu *et al.* revealed that women with pre-eclampsia had lower levels of LXA4, its receptor FPR2/ALX, and the enzymes involved in LXA4 production in their blood. To explore the connection between LXA4 and pre-eclampsia, experimental rats were treated with LXA4. The treated rats showed improvements in pre-eclampsia symptoms, reduced levels of LPS-induced pro-inflammatory cytokines, and an increase in the anti-inflammatory cytokine IL-10. Additionally, it was found that rats with blocked LXA4-signaling pathways developed pre-eclampsia-like symptoms ( Xu *et al.* 2014). Taken together, these two studies offer conflicting findings on the role of lipoxins in the regulation of pre-eclampsia. Future research examining lipoxin production over time, along with further analysis of receptors and enzyme-related products, could help clarify how this pathway is dynamically regulated in women with pre-eclampsia ( Elliott *et al.* 2017).

Furthermore, anti-angiogenic role of LXA4 has been demonstrated in vitro on human umbilical vein endothelia cells (HUVECS) as well as a LXA4-dependent reduction of lipopolysaccaride (LPS)-induced endothelial hyperpermeability ( Liu *et al.* 2009, Pang *et al.* 2011). Besides, the synthetic analogue of LXA4, 5(S),6(R)-7-trihydroxymthyl heptanoate (BML-111), reduced systolic blood pressure, 24h urinary albumin excretion, serum TNFα, IL-8 levels, and LPS- dependent morphologic injury of kidney and placenta ( Lin *et al.* 2012). In summary, these findings highlight the potential role of SPM supplementation for prevention of PE and resulting preterm birth. Further studies are necessary to expand these findings and explore the distinct immune interactions involved in maternal-fetal biology.

## 7. First compounds with an enriched marine oil supplement on the market

In some multivitamin formulations, 30 mg of the described marine oil enriched formulation have been introduced to simulate the physiological amount of pro-resolving lipid mediators of the placenta. The first studies of this nutritional supplement have demonstrated its effectiveness in raising SPMs in plasma in different physiological and pathological conditions.

Having been able to detect a large deficit of SPMs in conditions of inflammation and as described in the protocol, it is estimated that the application of this new formulation will substantially improve both the level of SPMs in plasma and serum and the ratio between SPMs and prostaglandins.

In the studies of Elajami *et al.* (2016) and Souza *et al.* (2020) it was possible to see that the ideal doses in the intakes are between 1500 mg and 3000 mg. Elajami et al. used one formulation for a period of one year while Souza *et al.* used the formulation up to 24 hours. Commune to both studies was zero incidence of side effects, and the substantial increase in SPMs.

The eventual impact of these formulations on the occurrence of obstetrical complications such as pre-term birth, pre-eclampsia chorioamnionitis or amniotic inflammation remains to be evaluated. So far, no clinical studies have been conducted addressing these issues. However, the application of SPMs in the form of enriched marine oil supplements represents a promising concept for the reduction of inflammatory conditions in pregnant women and thus a first step in the creation of an immunological homeostasis.

## 8. Conclusions

In conclusion, the utilization of selective pro-resolving mediators, including monohydroxylates, holds promise for the management of various acute and chronic diseases across a wide range of medical conditions. Particularly in obstetrics, supplementation with enriched marine oil nutritional products shows potential for attenuating serious complications such as pre-eclampsia and preterm birth. However, further research is necessary to determine optimal dosing regimens and fully elucidate the mechanisms underlying their therapeutic effects. Moreover, data from several studies indicate that intake of SPMs might have a different impact on high-risk vs. low-risk pregnancies, since their expression varies with progression of e.g. PE, Therefore, evidence suggests that dividing patients into risk groups may be necessary to thoroughly explore the potential of SMPs during pregnancy. Additionally, well-controlled clinical trials are essential to assess the effectiveness of SPM supplementation at various stages of high-risk pregnancies. Nonetheless, the findings suggest that these interventions may represent a valuable approach for addressing inflammatory diseases and mitigating obstetrical complications, thereby improving maternal and fetal health outcomes.

## Data Availability

No data are associated with this article.
