# Supplementary material for: Omega-3 long chain fatty acids and their metabolites in pregnancy outcomes for the modulation of maternal inflammatory- associated causes of preterm delivery, chorioamnionitis and preeclampsia
Source: F1000Res. 2024 Dec 23;13:882. Originally published 2024 Aug 5. [Version 3] doi: 10.12688/f1000research.153569.3 (PMC11809487; doi:10.12688/f1000research.153569.3)
Supplement: Peer review history

# Reviewer report: Reviewer response for version 3

**DOI:** 10.5256/f1000research.176157.r351973

**Published:** 2025-02-08

**Authors:** Susan Carlson

**Affiliations:** 1 Department of Dietetics and Nutrition, University of Kansas Medical Center, Kansas, KS, United States Virgin Islands

I am fine with revisions.

Is the review written in accessible language?

Yes

Are all factual statements correct and adequately supported by citations?

Partly

Are the conclusions drawn appropriate in the context of the current research literature?

Partly

Is the topic of the review discussed comprehensively in the context of the current literature?

Partly

Reviewer Expertise:

Preterm birth and omega-3 fatty acids.

I confirm that I have read this submission and believe that I have an appropriate level of expertise to confirm that it is of an acceptable scientific standard.

# Reviewer report: Reviewer response for version 2

**DOI:** 10.5256/f1000research.173064.r339291

**Published:** 2024-12-03

**Authors:** Susan Carlson

**Affiliations:** 1 Department of Dietetics and Nutrition, University of Kansas Medical Center, Kansas, KS, United States Virgin Islands

The authors briefly summarize the evidence for omega-3 fatty acids in maternal preterm delivery, but most of the paper is on inflammation and resolution of inflammation.  I've given some suggestions for more recent work on this subject that they do not seem to be aware of. I do think the title should better represent what is discussed. For example, marine oil supplements should be replaced by omega-3 long chain fatty acids. Also, the sections on chorioamnionitis and preeclampsia should follow the section on preterm birth and the title should say pregnancy outcomes.

 Please reference guidelines for omega-3 fatty acid supply in pregnancy for risk reduction of preterm and early preterm birth in addition to Koletzko et al in section 2. The guidelines reflect recent work that recognizes those who need higher dose DHA supplementation to reduce PTB and early PTB.*

 No need to say the results "suggest" with regard to the Cochrane Review. The results show benefit. 

 Carlson et al. found a reduction in PTB with high dose DHA, but the results also were notable in that participants with low baseline DHA status benefited from high dose DHA with lower early PTB (<34 wks).  

 I suggest omitting the statement attributed to Simmonds et al. on increased risk with high dose DHA. The authors state "relative risk." in that manuscript. While I don't dispute their finding, the prevalence of early PTB and blood levels achieved in the high dose group were similar to the 200 mg in Carlson et al. - meaning adherence to the high dose was poor. This has been pointed out by Jackson and Harris (PMID:32390621).

Is the review written in accessible language?

Yes

Are all factual statements correct and adequately supported by citations?

Partly

Are the conclusions drawn appropriate in the context of the current research literature?

Partly

Is the topic of the review discussed comprehensively in the context of the current literature?

Partly

Reviewer Expertise:

Preterm birth and omega-3 fatty acids.

I confirm that I have read this submission and believe that I have an appropriate level of expertise to confirm that it is of an acceptable scientific standard, however I have significant reservations, as outlined above.

## References

[1] : Omega-3 fatty acid supply in pregnancy for risk reduction of preterm and early preterm birth. *Am J Obstet Gynecol MFM* .2024;6(2) : 10.1016/j.ajogmf.2023.101251 101251 10.1016/j.ajogmf.2023.101251 38070679

[2] : Photobiomodulation for Alzheimer's Disease: Translating Basic Research to Clinical Application. *J Alzheimers Dis* .2020;75(4) : 10.3233/JAD-191210 1073-1082 10.3233/JAD-191210 32390621 PMC7369090

# Response

**Published:** 2024-12-18

**Authors:** Pedro Antonio Regidor

**Affiliations:** Medical Department, Exeltis Healthcare, Ismaning, Baviera, Germany

Thanks to the reviewer for the very valuable comments.

 We have changed the title of the review according to the suggestions.

 And we have included a statement regarding the new guideline for the supplementation of Omega-3 fatty acids in pregnancy to reduce preterm ean early preterm birth.

# Response

**Published:** 2024-12-23

**Authors:** Pedro Antonio Regidor

**Affiliations:** Medical Department, Exeltis Healthcare, Ismaning, Baviera, Germany

We have introduced all suggested changes.

 We have also adapted the title and the literature. 

 These changes , suggested by the reviewer, have also be introduced in this new version of the article.

# Reviewer report: Reviewer response for version 2

**DOI:** 10.5256/f1000research.173064.r336667

**Published:** 2024-11-04

**Authors:** Carol Wang

**Affiliations:** 1 The University of Newcastle, Callaghan, New South Wales, Australia; 2 Mothers' and Babies' Research Program, Hunter Medical Research Institute, Newcastle, New South Wales, Australia

The manuscript is significantly improved and I have no further comments to add.

 Well done!

Is the review written in accessible language?

Yes

Are all factual statements correct and adequately supported by citations?

Partly

Are the conclusions drawn appropriate in the context of the current research literature?

Partly

Is the topic of the review discussed comprehensively in the context of the current literature?

Partly

Reviewer Expertise:

Statistician/Bioinformatician. I have co-supervised (with my clinician supervisor) a student who performed a comprehensive review of Omega-3 supplementation and its role to mitigate preterm birth.

I confirm that I have read this submission and believe that I have an appropriate level of expertise to confirm that it is of an acceptable scientific standard.

# Reviewer report: Reviewer response for version 1

**DOI:** 10.5256/f1000research.168487.r312357

**Published:** 2024-08-24

**Authors:** Carol Wang

**Affiliations:** 1 The University of Newcastle, Callaghan, New South Wales, Australia; 2 Mothers' and Babies' Research Program, Hunter Medical Research Institute, Newcastle, New South Wales, Australia

The authors have provided a review of the potential role of SPMs in the mitigation of adverse pregnancy outcomes. In particular, they have addressed the possible mechanism involved and its potential to mitigate pre-eclampsia and preterm birth.

 Introduction/Background

 The authors have provided a summary of preterm birth, covering mainly its short-term consequences. The authors should consider expanding on the background and address the medium to long-term consequences as well.

 Supplementation with PUFAs to prevent preterm birth

 In this section, the authors described the role of PUFAs in preventing preterm birth, highlighting recommended guidelines, potential mechanisms that help with mitigating preterm birth, and the benefits of consuming omega-3-rich products.

 I noticed the authors specified the 2018 Cochrane review to be the latest Cochrane review, are they aware of the updated review that included RCTs published to August 2021 (Best et al.;2022  Ref 1)? Please review the updated Cochrane review and update the statements in this section accordingly.

 Additionally, the authors stated an increasing need to further elaborate the role of omega-3 long chain PUFAs on PTB concerning dosing, timing of supplementation and testing of baseline status, which was again mentioned in the conclusion but had not provide coverage regarding the existing literature. There are several publications available and the authors should review the literature and address these aspects accordingly. These are the PMIDs of some of the literature in the field that addressed dosing and the importance of testing before initiating supplementation: Ref 11 **,** Ref2 , Ref 3 , Ref 4, Ref 5 

 Pre-eclampsia

 The pre-eclampsia section is brief compared to the other sections of the study even though it is supposedly one of the specified outcomes the authors wished to address. They have presented the potential mechanisms but had not reviewed the evidence in the literature. Both animal studies and human studies (Ref 6, Ref 7,  Ref 8) have been presented regarding plasma LXA4 (and beyond); the authors should consider the literature and expand on the PE section. The authors might also want to consider examining the literature around the efficacy of  omega-3 supplementation and PE (Ref 9, Ref 10). In addition, the authors have not mentioned that PE often results in medically indicated PTB, as the resolution of PE is the delivery of the baby and the placenta.     

 Conclusions

 The authors presented promising evidence that SPMs could mitigate adverse outcomes in pregnancy. It would be worthwhile to perform a more thorough search of the literature and address the potential importance of stratifying the population accordingly (high-risk vs low-risk pregnancies, and baseline levels) and supplementation.

Is the review written in accessible language?

Yes

Are all factual statements correct and adequately supported by citations?

Partly

Are the conclusions drawn appropriate in the context of the current research literature?

Partly

Is the topic of the review discussed comprehensively in the context of the current literature?

Partly

Reviewer Expertise:

Statistician/Bioinformatician. I have co-supervised (with my clinician supervisor) a student who performed a comprehensive review of Omega-3 supplementation and its role to mitigate preterm birth.

I confirm that I have read this submission and believe that I have an appropriate level of expertise to confirm that it is of an acceptable scientific standard, however I have significant reservations, as outlined above.

## References

[1] : The role of omega-3 polyunsaturated fatty acids in the prevention of preterm birth. *Med J Aust* .2024;220(10) : 10.5694/mja2.52301 502-504 10.5694/mja2.52301 38714366

[2] : Plasma Concentrations of Long Chain N-3 Fatty Acids in Early and Mid-Pregnancy and Risk of Early Preterm Birth. *EBioMedicine* .2018;35: 10.1016/j.ebiom.2018.07.009 325-333 10.1016/j.ebiom.2018.07.009 30082226 PMC6156714

[3] : Omega-3 fatty acid supplementation in pregnancy-baseline omega-3 status and early preterm birth: exploratory analysis of a randomised controlled trial. *BJOG* .2020;127(8) : 10.1111/1471-0528.16168 975-981 10.1111/1471-0528.16168 32034969

[4] : Higher dose docosahexaenoic acid supplementation during pregnancy and early preterm birth: A randomised, double-blind, adaptive-design superiority trial. *EClinicalMedicine* .2021;36: 10.1016/j.eclinm.2021.100905 100905 10.1016/j.eclinm.2021.100905 34308309 PMC8257993

[5] : ISSFAL statement number 7 - Omega-3 fatty acids during pregnancy to reduce preterm birth. *Prostaglandins Leukot Essent Fatty Acids* .2022;186: 10.1016/j.plefa.2022.102495 102495 10.1016/j.plefa.2022.102495 36228573

[6] : Omega-3 Long-Chain Polyunsaturated Fatty Acids and Preeclampsia: Trials Say "No," but Is It the Final Word?. *Nutrients* .2017;9(12) : 10.3390/nu9121364 10.3390/nu9121364 29244779 PMC5748814

[7] : Efficacy of n-3 fatty acids supplementation on the prevention of pregnancy induced-hypertension or preeclampsia: A systematic review and meta-analysis. *Taiwan J Obstet Gynecol* .2020;59(1) : 10.1016/j.tjog.2019.11.002 8-15 10.1016/j.tjog.2019.11.002 32039806

[8] : Expression of lipoxin A4, TNFα and IL-1β in maternal peripheral blood, umbilical cord blood and placenta, and their significance in pre-eclampsia. *Hypertens Pregnancy* .2014;33(4) : 10.3109/10641955.2014.931419 449-56 10.3109/10641955.2014.931419 24960456

[9] : The role of specialized pro-resolving mediators in maternal-fetal health. *Prostaglandins Leukot Essent Fatty Acids* .2017;126: 10.1016/j.plefa.2017.09.017 98-104 10.1016/j.plefa.2017.09.017 29031403 PMC5647871

[10] : Two trans-acting regulatory genes (vir and mod) control antigenic modulation in Bordetella pertussis. *J Bacteriol* .1988;170(11) : 10.1128/jb.170.11.5059-5066.1988 5059-66 10.1128/jb.170.11.5059-5066.1988 2903140 PMC211571

[11] : Corrigendum to 'Plasma concentrations of long chain N-3 fatty acids in early and mid-pregnancy and risk of early preterm birth'. *EBioMedicine* .2020;51: 10.1016/j.ebiom.2019.102619 102619 10.1016/j.ebiom.2019.102619 31927481 PMC6956750

[12] : Preeclampsia is associated with a deficiency of lipoxin A4, an endogenous anti-inflammatory mediator. *Fertil Steril* .2014;102(1) : 10.1016/j.fertnstert.2014.03.056 282-290.e4 10.1016/j.fertnstert.2014.03.056 24796762

[13] : Identifying women who may benefit from higher dose omega-3 supplementation during pregnancy to reduce their risk of prematurity: exploratory analyses from the ORIP trial. *BMJ Open* .2023;13(4) : 10.1136/bmjopen-2022-070220 e070220 10.1136/bmjopen-2022-070220 37068907 PMC10111924

# Response

**Published:** 2024-10-05

**Authors:** Pedro Antonio Regidor

**Affiliations:** Medical Department, Exeltis Healthcare, Ismaning, Baviera, Germany

PD Dr. Pedro-Antonio Regidor Medical Director Exeltis Europe & Global Projects

 Exeltis Germany GmbH Adalperostraße 84

 85737 Ismaning (Germany) E-Mail: pedro-antonio.regidor@exeltis.com

 F1000 Research

research@f1000.com

 04 October 2024

 Re: Manuscript submission "Enriched marine oil supplements in pregnancy for the modulation of maternal inflammatory- associated causes of preterm delivery" (Manuscript DOI: https://doi.org/10.12688/f1000research.153569.1)

 Dear Sir or Madam,

 Thank you for the opportunity to revise and resubmit our manuscript entitled “Enriched marine oil supplements in pregnancy for the modulation of maternal inflammatory- associated causes of preterm delivery” to *F1000 Research*.

 We once again appreciate the careful review and constructive suggestions. Following this letter are the reviewer’s comments with our responses, including how and where the text was modified. 

 We believe the revised manuscript addresses all reviewer’s comments and have met the reviewer's needs and concerns. The revision has been developed in consultation with all coauthors, and each author has given approval to the final form of this revision.

 This manuscript is original, has not been published before and is not currently being considered for publication elsewhere. All co-authors have read the manuscript and approved its submission to *F1000 Research*

 On behalf of all coauthors sincerely yours,

 PD Dr. Pedro-Antonio Regidor

 Comments raised by the reviewers and responses-

**REVIEWER’S COMMENT (Carol Wang)**

 The authors have provided a review of the potential role of SPMs in the mitigation of adverse pregnancy outcomes. In particular, they have addressed the possible mechanism involved and its potential to mitigate pre-eclampsia and preterm birth.

*Introduction/Background*

**Reviewer** **Comment #1:**

 The authors have provided a summary of preterm birth, covering mainly its short-term consequences. The authors should consider expanding on the background and address the medium to long-term consequences as well.

**Author Response #1:**

 Thank you for the remark. We added more information on long-term consequences of preterm birth in adulthood, which will further emphasize the burden of this health condition.

*Supplementation with PUFAs to prevent preterm birth*

 In this section, the authors described the role of PUFAs in preventing preterm birth, highlighting recommended guidelines, potential mechanisms that help with mitigating preterm birth, and the benefits of consuming omega-3-rich products.

**Reviewer Comment #2:**

 I noticed the authors specified the 2018 Cochrane review to be the latest Cochrane review, are they aware of the updated review that included RCTs published to August 2021 (Best et al.;2022 Ref 1)? Please review the updated Cochrane review and update the statements in this section accordingly.

**Author Response #2:**

 Thank you for pointing this out. We reviewed the updated review and included its findings in the text accordingly.

**Reviewer** **Comment #3:**

 Additionally, the authors stated an increasing need to further elaborate the role of omega-3 long chain PUFAs on PTB concerning dosing, timing of supplementation and testing of baseline status, which was again mentioned in the conclusion but had not provide coverage regarding the existing literature. There are several publications available and the authors should review the literature and address these aspects

 accordingly. These are the PMIDs of some of the literature in the field that addressed dosing and the importance of testing before initiating supplementation: Ref 11, Ref2 , Ref 3 , Ref 4, Ref 5

**Author** **Response #3:**

 We agree and are thankful for these suggestions which we included accordingly. We are convinced that this will add to a better understanding regarding possible dosages of omega-3 long chain PUFAs

*Pre-eclampsia*

**Reviewer Comment #4:**

 The pre-eclampsia section is brief compared to the other sections of the study even though it is supposedly one of the specified outcomes the authors wished to address. They have presented the potential mechanisms but had not reviewed the evidence in the literature. Both animal studies and human studies (Ref 6, Ref 7, Ref 8) have been presented regarding plasma LXA4 (and beyond); the authors should consider the literature and expand on the PE section. The authors might also want to consider examining the literature around the efficacy of omega-3 supplementation and PE (Ref 9, Ref 10). In addition, the authors have not mentioned that PE often results in medically indicated PTB, as the resolution of PE is the delivery of the baby and the placenta.

**Author** **Response #4:**

 Thank you for your comment and for providing relevant literature. We agree and included the main findings of the publications and think that this highly improves the manuscript.

*Conclusions*

**Reviewer** **Comment #5:**

 The authors presented promising evidence that SPMs could mitigate adverse outcomes in pregnancy. It would be worthwhile to perform a more thorough search of the literature and address the potential importance of stratifying the population accordingly (high-risk vs low-risk pregnancies, and baseline levels) and supplementation.

**Author Response #5:**

 Thank you for raising this comment. The conclusion has been adapted accordingly.
